# Heteroxanthin as a pigment biomarker for *Gonyostomum semen* (Raphidophyceae)

**DOI:** 10.1371/journal.pone.0226650

**Published:** 2019-12-18

**Authors:** Camilla Hedlund Corneliussen Hagman, Thomas Rohrlack, Silvio Uhlig, Vladyslava Hostyeva

**Affiliations:** 1 Limnology and Hydrology group, Section for Soil and Water, Faculty of Environmental Sciences and Natural Resource Management, Norwegian University of Life Sciences, Ås, Norway; 2 Toxinology Research Group, Norwegian Veterinary Institute, Oslo, Norway; 3 Norwegian Culture Collection of Algae, Section for Microalgae, Norwegian Institute for Water Research, Oslo, Norway; University of Siena, ITALY

## Abstract

The ability to identify drivers responsible for algal community shifts is an important aspect of environmental issues. The lack of long-term datasets, covering periods prior to these shifts, is often limiting our understanding of drivers responsible. The freshwater alga, *Gonyostomum semen* (Raphidophyceae), has significantly increased distribution and mass occurrences in Scandinavian lakes during the past few decades, often releasing a skin irritating slime that causes discomfort for swimmers. While the alga has been extensively studied, long-term data from individual lakes are often absent or greatly limited and drivers behind this species’ success are still not clear. However, if specific and persistent taxa biomarkers for *G*. *semen* could be detected in dated sediment cores, long-term data would be improved and more useful. To test for biomarkers, we examined the pigment composition of several *G*. *semen* strains in culture. Further, dated sediment core samples from Lake Lundebyvann, Norway, were used to test the pigments’ suitability as biomarkers in paleolimnological studies. Modifications to a common analysis allowed for the successful detection of the polar xanthophyll heteroxanthin and the non-polar chlorophyll *a*, as well as several other algal pigments by using high performance liquid chromatography-photometric diode arrays (HPLC-PDA). Heteroxanthin was confirmed by liquid chromatography-mass spectrometry (LC-MS) and detected by HPLC-PDA in all examined *G*. *semen* strains, along with chlorophyll *a*. Using HPLC-PDA, we also identified and confirmed the presence of the biomarker, xanthophyll heteroxanthin, in sediment core samples up to 60 years of age. The specificity of this xanthophyll was also tested by examining a wide range of algal strains from common Norwegian phytoplankton species. Heteroxanthin was not detected in any species commonly occurring in significant amounts in Norwegian lakes. We therefore conclude that heteroxanthin is a suitable pigment biomarker for *G*. *semen* and that this pigment can be successfully used for paleolimnological studies.

## Introduction

Studies involving microalgae and in particular, algal blooms, have been increasing worldwide over several decades. One example is in northern Europe on the freshwater Raphidophyte *Gonyostomum semen*. This slime-producing flagellate first appeared in large biomasses in Scandinavian lakes in the late 1940s (Sweden) and 1970s (Norway and Finland) with reports of occurrence of skin irritations for swimmers. The alga was discovered in an increasing number of lakes [1–5]. This increasing trend has continued [6, 7]. Yet the drivers of this expansion remained largely unknown despite a considerable number of studies on this topic [[6–9] among others]. The main challenge in describing these drivers is in part due to the lack of long-term datasets. In Norway, in particular, monitoring of phytoplankton started in 1970s, therefore data describing periods prior to the expansion of *G*. *semen* are lacking. Although this species appears in a wide range of lake types, *G*. *semen* mass occurrences are usually associated with humic lakes with low nutrient concentrations and low pH [3, 4, 6, 9–11]. Unfortunately, these lake types have not been a focal point of monitoring efforts.

The combined use of historical algal records and environmental factors might be useful in explaining the expansion of *G*. *semen*. Previous studies have tried to detect *G*. *semen* cysts in lake sediments by means of qPCR, however the survival time of the cysts in sediments is not known [12]. Environmental DNA specific to *G*. *semen* has been successfully detected in water samples, although not completely species specific [6]. No efforts have been made for detecting *G*. *semen* eDNA in sediment cores, a material that is typically more difficult for DNA extraction than, for instance, water samples. Pigments preserved in lake sediments however, have already been widely used to reconstruct long-term changes in lake production, algae community composition, eutrophication, acidification and climate change [[13–15] and [16] and references therein]. These methods are well established, as is also the use of pigment biomarkers [e.g. [17–19]]. All photosynthetic organisms contain chlorophyll *a*, and in addition a mixture of other chlorophylls (*b*, *c1*, *c2*), and photo protective pigments (carotenoids) such as xanthophylls and carotenes. These pigments can represent different taxonomic groups or algal classes, and differ in their specificity [16, 17]. Bulk sediment samples contain a cocktail of such pigments in varying compositions due to the presence of a variety of sedimented algal species. If pigments representative for *G*. *semen* could be identified in sediments, they could be used to reconstruct the expansion of *G*. *semen*.

The pigment composition of *G*. *semen* has been studied on several occasions, starting with the discovery of chlorophyll *a* and four unidentified carotenoids in natural samples in 1966 [20]. Studies on pure cultures led to the additional detection of three xanthophylls, one carotenoid (presumably β-carotene) and chlorophyll *c* [21], later supplemented by identification of the xanthophylls diadinoxanthin, dinoxanthin, neoxanthin, heteroxanthin and the carotene β-β by Fiksdahl et al. [22]. Recently, Sassenhagen et al. [23] discovered that *G*. *semen* cultures also contained small amounts of violaxanthin and zeaxanthin, in addition to significant amounts of alloxanthin. Most of these pigments occur in many phytoplankton groups and are therefore not suitable as biomarkers for quantifying *G*. *semen*. Alloxanthin is only found as a major pigment in Cryptophytes [24], however this algae group often co-occurs along with *G*. *semen*, at times in large amounts. Therefore, this pigment is also unsuitable as a biomarker for *G*. *semen* quantification. Heteroxanthin is a polar pigment that was originally discovered as an unknown xanthophyll in the terrestrial species *Vaucheria sessilis* and *Botrydium granulatum* (Xanthophyceae) [25]. The pigment was later found in another *Vaucheria* sp. and named heteroxanthin by Strain et al. [26]. Later, heteroxanthin was discovered in species of *Tribonema* (Xanthophyceae) [[27] and reference in [28]] and in *Euglena gracilis* (Euglenophyceae) [28]. In 1984 this xanthophyll was detected in cultures of *G*. *semen* and *Vacuolaria virescens* (Raphidophyceae) [22], and later in *Phaeothamnion confervicola*, *Phaeoschizochlamys mucosa*, *Pleurochloridella botrydiopsis* and *Stichogloea doederleinii* (Phaeothamniophyceae) [29, 30]. These findings characterize heteroxanthin as a rare and minor pigment that occurs mainly in organisms that, except *G*. *semen*, seldom occur or dominate the phytoplankton population in Norwegian lakes.

Therefore, we tested the suitability of using heteroxanthin as a quantitative biomarker for *G*. *semen* in paleolimnological studies, using sediment cores as biological archives. We investigated the pigment composition of several *G*. *semen* strains to confirm the presence of heteroxanthin by high performance liquid chromatography-photometric diode array (HPLC-PDA) and liquid chromatography-mass spectrometry (LC-MS). We then confirmed the specificity of this pigment by examining the presence of heteroxanthin in algae cultures of species commonly co-occurring with *G*. *semen* in Norwegian lakes.

## Materials and methods

### Preparation of material

#### Algal cultures

In this study, 29 cultures of *G*. *semen* and 65 cultures from additional taxa, all deposited in the Norwegian Culture Collection of Algae (NORCCA), Oslo, were analyzed for pigment composition. A list of *G*. *semen* strains is given in Table 1. The other cultures were chosen to represent phytoplankton taxa typical for Norwegian lakes where *G*. *semen* also occurs. These included the classes Bacillariophyceae, Chlorophyceae, Chrysophyceae, Conjugatophyceae, Cryptophyceae, Cyanophyceae, Dinophyceae, Euglenophyceae, Mediophyceae, Phaeothamniophyceae, Raphidophyceae, Synurophyceae, Trebouxiophyceae and Xanthophyceae. In addition, cultures of phytoplankton species with a confirmed occurrence of heteroxanthin were used as reference material to compensate for the lack of commercial heteroxanthin standard. These include *Euglena gracilis* NIVA-1/79, synonym SAG 1224-5/25 as published in Nitsche [28], *Phaeothamnion confervicola* K-1186, synonym CCMP 637 published in Andersen et al. and Bailey et al. [29, 30], as well as one strain of *Tribonema aequale*. A full list of phytoplankton cultures other than *G*. *semen* is given in S1 Table.

**Table 1:** Strains of *Gonyostomum semen* analyzed for pigment composition.

Strain number	Origin
NIVA-7/05	Lake Vansjø (Grepperødfjorden, SE-Norway), 2005
NIVA-2/09	Lake Adalstjern (S-Norway)
NIVA-2/10	Lake Bökesjön (Sweden)
NIVA-5/13	Lake Langsæ Øst (S-Norway), 2012
NIVA-6/13	Lake Prestvatnet (SW-Norway), 2012
NIVA-10/13	Farm pond, Askim (SE Norway)
NIVA-11/13	Farm pond, Askim (SE Norway)
NIVA-12/13	Lake Bjørkelangen (SE Norway), 2013
NIVA-13/13	Lake Bjørkelangen (SE Norway), 2013
NIVA-15/13	Lake Bjørkelangen (SE Norway), 2013
NIVA-16/13	Lake Bjørkelangen (SE Norway), 2013
NIVA-17/13	Lake Bjørkelangen (SE Norway), 2013
NIVA-18/13	Lake Rødnessjøen (SE Norway), 2013
NIVA-24/13	Lake Vansjø (Nesparken, SE Norway), 2013
NIVA-33/13	Lake Mjöträsket (N-Sweden), 2010
NIVA-34/13	Lake Kylänalanen (Finland), 2010
K-1835	Arnh. Sloughs, Michigan (USA), 2011
NOR 17	Lake Brønnerødtjern (SE-Norway), July 2018
NOR 18	Lake Brønnerødtjern (SE-Norway), July 2018
NOR 19	Lake Brønnerødtjern (SE-Norway), Sept. 2018
NOR 20	Lake Brønnerødtjern (SE-Norway), Sept. 2018
NOR 21	Lake Brønnerødtjern (SE-Norway), Sept. 2018
NOR 22	Lake Brønnerødtjern (SE-Norway), Sept. 2018
NOR 23	Lake Brønnerødtjern (SE-Norway), Sept. 2018
NOR 24	Lake Brønnerødtjern (SE-Norway), Sept. 2018
NOR 25	Lake Brønnerødtjern (SE-Norway), Sept. 2018
NOR 26	Lake Brønnerødtjern (SE-Norway), Sept. 2018
NOR 27	Lake Brønnerødtjern (SE-Norway), Sept. 2018

*Gonyostomum semen* strains analyzed for pigment composition using high performance liquid chromatography-photometric diode array, listed with Norwegian Culture Collection of Algae strain numbers and strain origin.

*G*. *semen* cultures were grown at 20°C with a light:dark cycle of 14:10 and light intensity of 20 μmol m^2^ s^-1^. They were harvested 14–21 days after last inoculation. The cultures were microscopically examined to ensure the cells were growing in a healthy shape, without cysts or broken cells. Strain NIVA-2/09 was harvested after five weeks due to poor growth. The density of the cultures at harvest was variable, and correct measurements of dry weight was not possible due to low biomass and variable mucilage content which contributed to the dry weight. Cultures of other taxa were grown at 16°C with light intensity at 5–10 μmol m^2^ s^-1^ and a light:dark cycle of 16:8. Harvesting was done during variable growth phases and densities. Sample sizes varied. Either 20–30 ml of *G*. *semen* cultures or 8–10 ml of other taxa were filtered onto GF/C filters and immediately frozen (-20°C) in individual 15 ml Falcon tubes prior to freeze drying and analysis (see below for methods). Following this step, the samples were protected from light at all times.

#### Sediment core sampling

Sediment core samples were retrieved by using a Uwitec core sampler (diameter 6 cm). The core was sliced into 1 cm samples, placed in individual airtight containers and frozen and protected from light until freeze-dried. The top cm of the core was excluded due to high content of water on the sediment surface. Freeze-drying was performed within 2–4 weeks after sampling and immediately before extraction. Dating of the core sections was performed using Pb^210^ dating on a separate core from the same lake, as published by Xiao et al. [31].

#### Pigment extraction

Pigments were extracted directly from the freeze-dried algal culture filters and from 250 mg (+/-50 mg) freeze-dried sediment samples by adding 3 ml of 100% acetone containing 1 μg ml^-1^ β-apocarotenal (Sigma-Aldrich, Oslo, Norway) as an internal standard. For sediment samples with a suspected low pigment content, the extracted amount of sediment was increased to 1000 mg (±50 mg). All extracts were vortexed and extracted for 24hours in the dark at 0–4°C. Thereafter, the filters from culture samples were removed, and all sample were centrifuged at 3000 rpm for 10 minutes. The supernatant was transferred to HPLC vials. Water was added at a final concentration of 20% to improve separation of polar pigments. Analysis was performed within 48 hours. The extracts were kept cool and dark during the entire process.

### Pigment analysis using HPLC-PDA

The HPLC-PDA analysis was performed on a Dionex^TM^ UltiMate 3000 HPLC (Thermo Scientific^TM^) with an Acclaim^™^ C30 column, 3 μm (Thermo Scientific^™^), using a modified procedure of the Wright et al. [32] method. Modifications included use of a column with smaller particles and a reduced flow rate set at 0.5 ml min^-1^. The solvents used were: A) 100% methanol; B) 90:10 acetonitrile (HPLC quality) and Milli-Q water; C) 100% ethyl acetate (HPLC quality); and D) 0.8 M ammonium acetate (HPLC quality). Ammonium acetate was increased from the original method to 0.8 M in order to enhance separation and sharpen the polar peaks. The procedure improvements, shown in Table 2, were necessary in order to enhance detection of the polar pigment heteroxanthin while still detecting non-polar chlorophyll *a*. This was achieved by increasing step 2 (100% solvent B) with eight minutes, and inserting a step 3 (20% B, 80% C) with a two minute flattened curve before two minutes of linear curve indicated in step 4 (Table 2). A 20 μl sample was injected for each run. The optical detector was set to monitor absorption between 350 and 700 nm. Peak quantification was determined at 436 nm and the internal standard was used to calibrate the system.

**Table 2:** Procedure used for high performance liquid chromatography (HPLC).

Step	Time (min)	Flow rate (ml min^-1^)	% A	% B	% C	% D	Curve
1	0	0.5	80	0	0	20	5
2	4	0.5	0	100	0	0	5
3	26	0.5	0	20	80	0	7
4	28	0.5	0	20	80	0	5
5	30	0.5	0	100	0	0	5
6	32	0.5	80	0	0	20	5
7	38	0.5	Stop				

HPLC procedure modified from Wright et al. [32]: Solvent A) 100% methanol; B) 90:10 acetonitrile:Milli-Q water; C) 100% ethyl acetate and D) ammonium acetate (0.8M). Solvents B, C, and D were HPLC quality grade.

Identification of pigments were performed manually using Dionex^TM^ Chromeleon^TM^ version 7.2.6 (Thermo Scientific^TM^). Pigment standards provided by DHI, (Hørsholm, Denmark) were used to identify pigments other than heteroxanthin in *G*. *semen* strains. Putative heteroxanthin was initially and tentatively identified in *G*. *semen* cultures according to Buchecker and Liaaen-Jensen [33] as a polar xanthophyll with absorption peaks at 423, 444 and 474 (+/-1) nm. The identification was substantiated by comparing chromatograms and peak absorption spectra with those of confirmed heteroxanthin producing strains. The identification of heteroxanthin was further verified by LC–HRMS analysis of the *G*. *semen* strain NIVA-17/13 to establish the accurate mass and elemental composition of the pigment (see below).

The HPLC-PDA analysis of sediment samples and samples of phytoplankton species other than *G*. *semen* focused on heteroxanthin and chlorophyll *a* only. Products of chlorophyll *a* breakdown occurring in sediment samples were identified according to their absorption spectra.

### Heteroxanthin LC–HRMS analysis

A fresh sample of the *G*. *semen* strain NIVA-17/13 was analyzed by chromatography using a 150 × 2.1 mm i.d. 2.6 μm Kinetex F5 column (Phenomenex, Torrance, CA, USA). The mobile phase (250 μl min^-1^) consisted of 5 mM ammonium formate (A), and 5 mM ammonium formate in 95:5 methanol-water (B). The column was eluted using a linear gradient from 70–100% B over 12 min, then flushed with 100% B for 2.5 min, followed by return to 70% B and equilibration with 70% B for 2.5 min using a Vanquish Horizon UHPLC pump (Thermo Fischer Scientific, Waltham, MA, USA). The mass spectrometer was a Q-Exactive Fourier-transform high-resolution mass spectrometer (Thermo Fischer Scientific) equipped with a heated electrospray ionization interface (HESI-II). The mass spectrometer was run in positive and negative ion full-scan modes using fast polarity switching (i.e., alternating positive and negative ion scans), in the mass range *m/z* 400–800. The mass resolution was set to 70,000 at *m/z* 200. The spray voltage was 4 kV, the transfer capillary temperature was 250°C, and the sheath and auxiliary gas flow rates were 35 and 10 units, respectively. Xcalibur 2.3 or 3.0 (Thermo Fisher Scientific) was used to calculate elemental compositions and mass errors of observed ions.

## Results

### Identification of *G*. *semen* pigments

**Fig 1:**
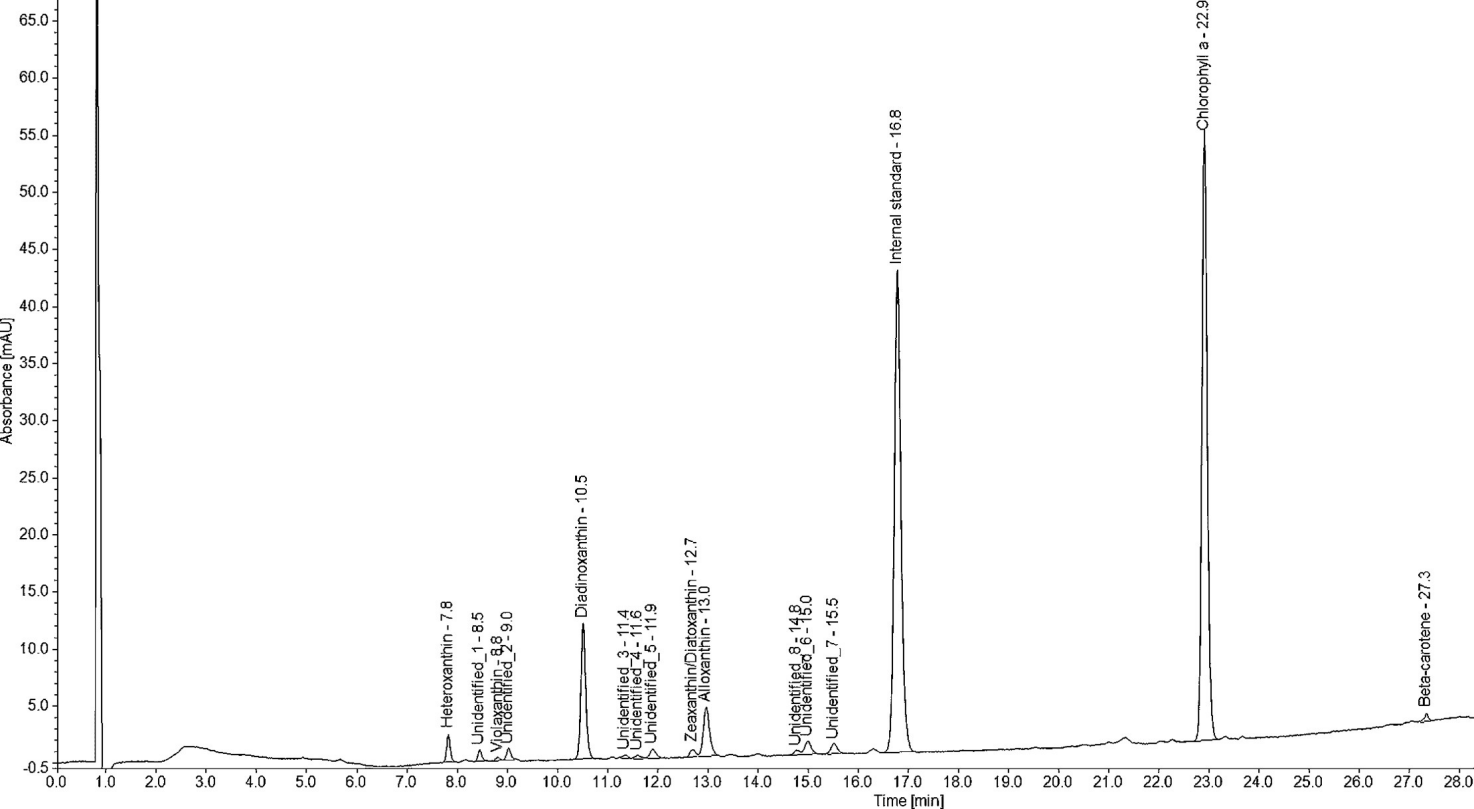
*Gonyostomum semen* chromatogram. The full chromatogram of strain NOR 20 from high performance liquid chromatography-photometric diode array. The x-axis gives the retention time (minutes) from the injection peak at 0 minutes to 28 minutes, y-axis gives absorbance as milli-Absorbance Units (mAU). Labels for each peak represents the pigment identification and retention time.

**Fig 2:**
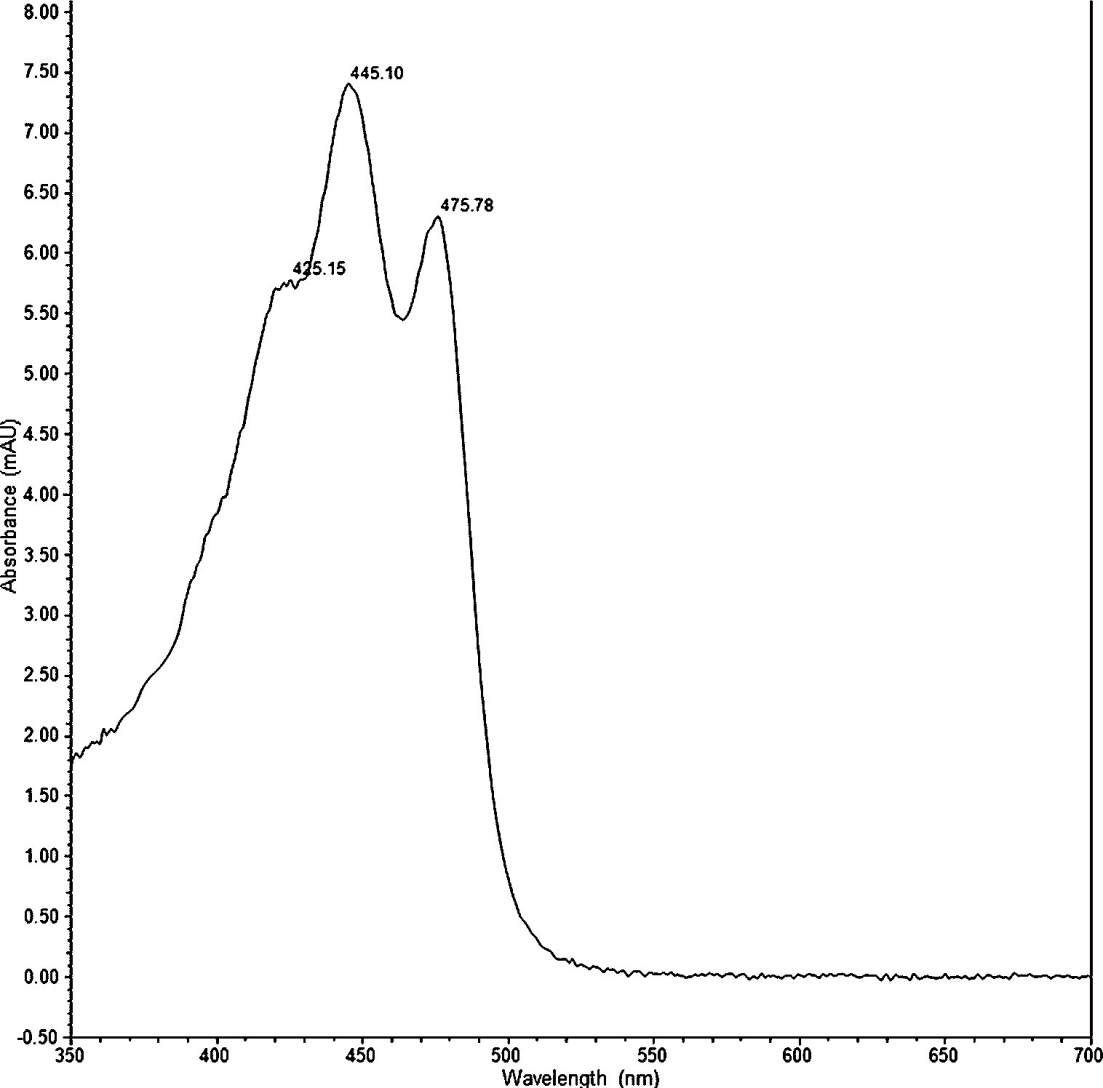
Heteroxanthin absorption spectrum. A typical absorption spectrum of xanthophyll heteroxanthin as detected in *Gonyostomum semen* strain NIVA-5/13 by high performance liquid chromatography-photometric diode array. X-axis gives wavelengths from 350–700 nm, y-axis gives absorbance (mAU). λ_max_ are seen at 425.15, 445.10 and 475.78 nm.

**Fig 3:**
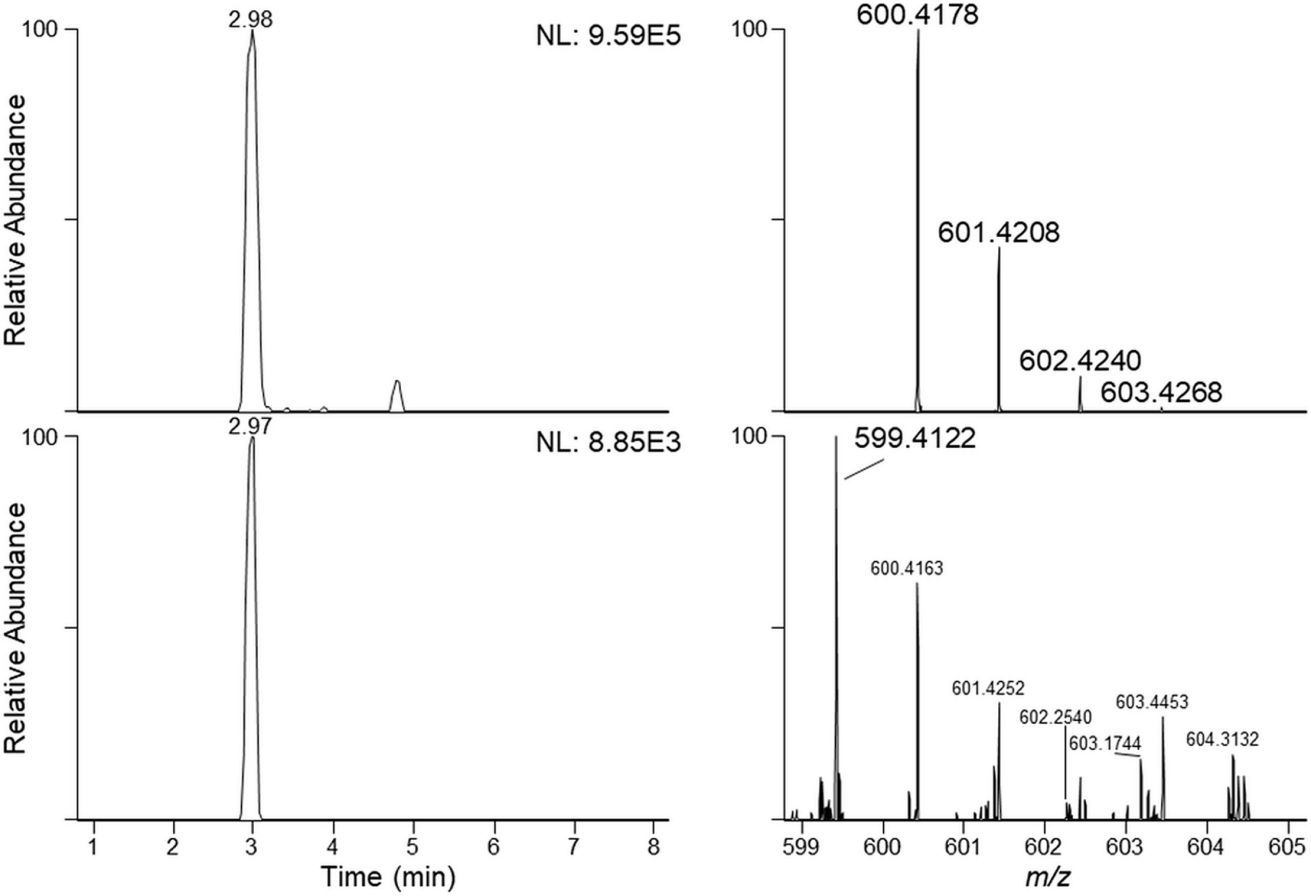
*Gonyostomum semen* extracted ion chromatograms and mass spectra. Extracted ion LC–HRMS chromatograms including full-scan mass spectra for M^+·^ (upper trace, ± 5 ppm) and [M−H]^−^ (lower trace, ± 5 ppm) of putative heteroxanthin in a fresh extract from *G*. *semen* strain NIVA-17/13. The number in the top right-hand corner of each chromatogram is the intensity of the highest peak in that chromatogram (arbitrary units).

Pigment identification by HPLC-PDA was successful for all *G*. *semen* cultures. A typical HPLC-PDA chromatogram of *G*. *semen* is shown in Fig 1. A xanthophyll pigment was detected in all *G*. *semen* strains at 7.8 min (±0.1) using the HPLC-PDA method. The xanthophyll had absorption spectrum λ_max_ at 425, 445 and 475 nm, as shown in Fig 2. The relatively short retention time and the absorption spectrum was in accordance with the expected and reported characteristics of heteroxanthin. The same peak was observed in chromatograms from phytoplankton species with a known ability to produce heteroxanthin (Table 3). Putative heteroxanthin in *G*. *semen* afforded ions of *m/z* 600.4178 and 599.4122 following electrospray ionization in the positive and negative ion mode, respectively (Fig 3). The elemental compositions of these ions were calculated to C_40_H_56_O_4_ (+1.75 ppm for a radical ion) and C_40_H_55_O_4_ (+0.03 ppm) for the principal positive and negative ions, respectively. These elemental formulae were in agreement with the radical ion (M^+**·**^) of heteroxanthin in positive ion mode, and with the deprotonated molecule ([M−H]^−^) of heteroxanthin in negative ion mode. Thus, the elemental formula of the neutral molecule was C_40_H_56_O_4_, which is the correct composition of heteroxanthin. The signal intensity of M^+**·**^ was approximately 100-fold higher compared to [M−H]^−^. A pentafluorophenylpropyl-particle was very well suited for retention and separation of xanthophyll pigments prior to mass spectrometric detection. This shows that such a particle would be an alternative to the commonly used C30 reversed-phase columns.

**Table 3:** Cultivated algae species with heteroxanthin.

Strain number	Class	Species	Heteroxantin:chlorophyll *a*
NIVA-1/79	Euglenophyceae	*Euglena gracilis*	0.014
NIVA-85/9	Unidentified	Unidentified	0.024
NIVA-1/15	Raphidophyceae	*Vacuolaria virescens*	0.076
NIVA-2/13	Raphidophyceae	*Vacuolaria virescens*	0.132
NIVA-3/14	Raphidophyceae	*Vacuolaria virescens*	0.027
NIVA-4/14	Raphidophyceae	*Vacuolaria virescens*	0.081
K-0087	Xanthophyceae	*Tribonema aequale*	0.085
K-0162	Xanthophyceae	*Tribonema minus*	0.023
K-0173	Xanthophyceae	*Tribonema regulare*	0.040
K-1003	Phaeothamniophyceae	*Phaeothamnion* sp.	0.026
K-1186	Phaeothamniophyceae	*Phaeothamnion confervicola*	0.042

Cultures other than *Gonyostomum semen* with detected heteroxanthin by high performance liquid chromatography-photometric diode array. Heteroxanthin is given as ratio to chlorophyll *a*.

The modified HPLC-PDA method and the C30 column ensured satisfactory separation of the most important xanthophylls of *G*. *semen* (heteroxanthin, diadinoxanthin and alloxanthin), which all appeared within the first 13 minutes of the analysis (Fig 1). These compounds were found in all *G*. *semen* cultures along with chlorophyll *a*. In three cultures, an additional xanthophyll peak was found at 12.7 min (Fig 1), which, according to retention time and absorption spectrum, may be either zeaxanthin or diatoxanthin. Furthermore, a derivate of chlorophyll *c2*, violaxanthin and β-carotene were detected in some cultures, but only in minor amounts. Eight unidentified peaks were also observed (Fig 1). Three of these (Unidentified 5, 6 and 7) were found in all strains except NIVA-17/13 and NIVA-33/13.

**Fig 4:**
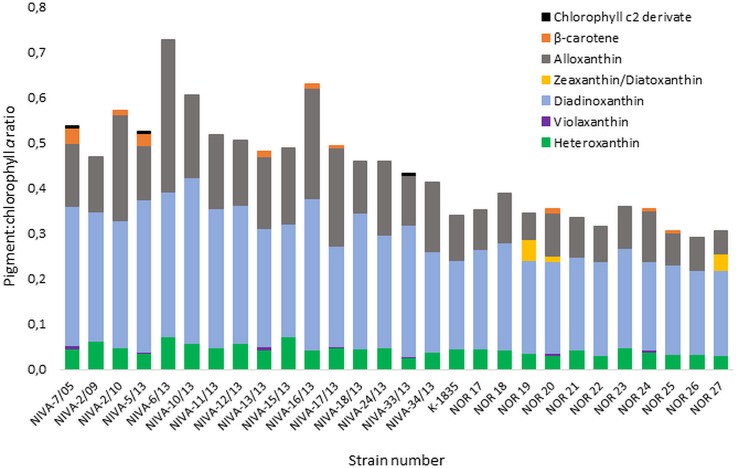
Pigment to chlorophyll *a* ratios of *Gonyostomum semen* cultures. The ratio (y-axis) of xanthophylls heteroxanthin, violaxanthin, diadinoxanthin, zeaxanthin or diatoxanthin, alloxanthin, carotene β-β, and derivate of chlorophyll *c2* in relation to chlorophyll *a* in all investigated strains of *G*. *semen* (x-axis) as detected by high performance liquid chromatography-photometric diode array.

The cultures showed great variability in pigment composition based on the pigment to chlorophyll *a* ratio, as shown in Fig 4. A full list of the ratios for all *G*. *semen* cultures can be found in S2 Table. The major accessory pigments identified in all cultures were diadinoxanthin (pigment:chlorophyll *a* ratios ranging from 0.185–0.367), alloxanthin (0.051–1.526) and heteroxanthin (0.026–0.073).

HPLC-PDA analysis of the additional phytoplankton taxa was successful for the majority of cultures tested, however some were excluded from further analysis due to no detectable pigments, including chlorophyll *a*. In addition to *G*. *semen* (Raphidophyceae), heteroxanthin was detected in four algal classes: Euglenophyceae (*Euglena gracilis* NIVA-1/79), Phaeothamniophyceae (*Phaeothamnion* sp. K-1003 and *P*. *confervicola* K-1186), Raphidophyceae (*Vacuolaria virescens* NIVA-1/15, NIVA2/13, NIVA-3/14 and NIVA-4/14) and Xanthophyceae (*Tribonema aequale* K-0087, *T*. *minus* K-0162 and *T*. *regulare* K-0173), as well as one unidentified strain (NIVA-85/9), as shown in Table 3. The heteroxanthin to chlorophyll *a* ratios ranged from 0.014 in *E*. *gracilis* to 0.132 in one strain of *V*. *virescens* (Table 3). Heteroxanthin was not detected in any other phytoplankton culture.

### Heteroxanthin as paleolimnological biomarker for *G*. *semen*

**Fig 5:**
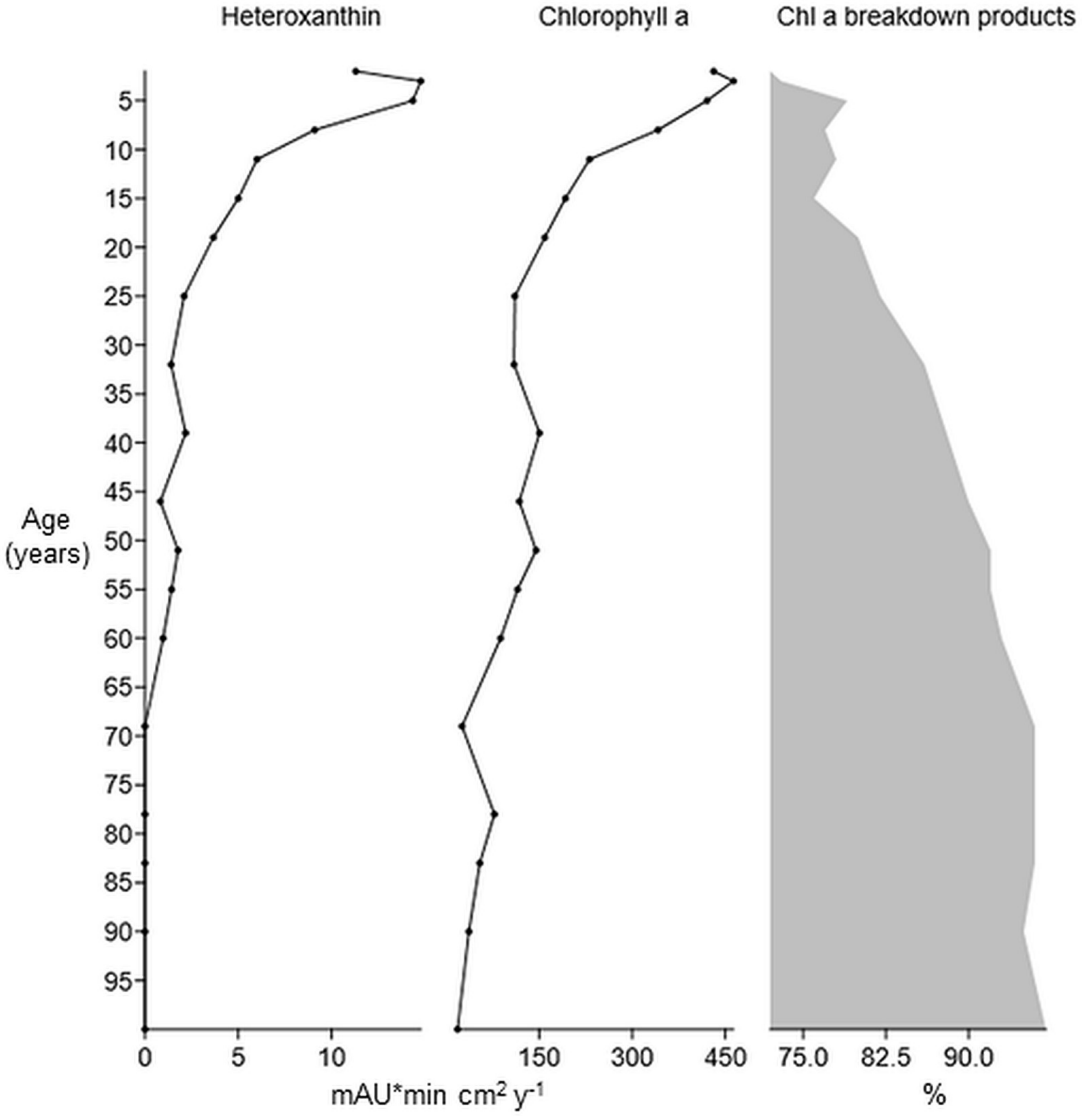
Stratigraphy of *Gonyostomum semen* pigments in sediment core samples. Amounts of heteroxanthin and total chlorophyll *a* (including breakdown products) in sediment core samples of age 2–100 years (y-axis) from lake Lundebyvann (SE-Norway), given as mAU*min cm^2^ y^-1^ (x-axis). The breakdown products share of total chlorophyll *a* is shown as percentage.

Heteroxanthin was successfully detected in sediment core samples corresponding to a maximum age of 60 years (+/- 13). A full HPLC-PDA chromatogram of an appr. 51 year old sediment sample, from which 1 g of sediment was extracted, and the associated absorption spectrum for heteroxanthin in this sample is given in S1 Fig and S2 Fig respectively. The detection parameters of heteroxanthin in sediment samples, including the similarity of the absorption spectrum compared to that of the reference material are given in S3 Table, showing that the manual identification of heteroxanthin is confirmed. The yearly amounts of heteroxanthin and chlorophyll *a* including breakdown products deposited in the lake sediments for the past 100 years are shown in Fig 5. In the samples age 50 and older, the heteroxanthin peak was found close to that of a chlorophyll *a* breakdown product. The clear differences in absorption spectra made the separation possible, however. Chlorophyll *a* increases from 1917 while the most pronounced increase occurs the latest 25 years. Heteroxanthin first appears 60 years ago, varying in amount the next 30 years, then rapidly increasing towards 2015. The most pronounced increase has occurred during the past 10 years for both heteroxanthin and chlorophyll *a*.

## Discussion

### Confirmation of pigment composition in *G*. *semen*

We detected several of the previously reported accessory pigments of *G*. *semen* by using a HPLC–PDA analytical method, complemented with LC–HRMS. The presence of heteroxanthin in *G*. *semen* culture NIVA-17/13 was initially assumed based on the HPLC–PDA data. Because of the lack of a reference standard for heteroxanthin, the identity of the compound was further investigated using LC–HRMS. HRMS supported the finding that xanthophyll was indeed heteroxanthin. The data showed that the elemental composition of the neutral molecule was C_40_H_56_O_4_ and thus in accordance with heteroxanthin, based on accurate mass measurements in the positive and negative ion modes (Fig 3). Using electrospray ionization in the positive mode, the compound afforded rather unusual radical cations. Even though the formation of radical ions during electrospray ionization is rare, it has been shown to occur for compounds with conjugated π-systems (so-called polyenes), e.g. β-carotene [34]. Although it appears from these earlier studies that the ratio between M^+**·**^ and [M+H]^+^ ions can be modulated by different solvents and by applying different source voltages, we did not study this for putative heteroxanthin. We also acquired HRMS/MS spectra from higher-energy collision dissociation of M^+**·**^. However, fragmentation of the heteroxanthin radical cations merely gave a myriad of low-intensity product ions, and the intensity of the deprotonated molecule in negative ion mode was too low for HRMS/MS. The peak in the polar region of the HPLC-PDA chromatogram (7.8 min) agreed with the hydrophilic nature of this pigment, and the absorption spectrum λ_max_ (acetone) of (420), 445 and 475 (+/-1) agreed with the expected λ_max_ (EtOH) of 444 and 474 nm [26, 33]. By using this modified HPLC-PDA procedure, the xanthophyll identified as heteroxanthin was found in all 29 cultures of *G*. *semen*, and as expected in all other algae strains previously reported as containing heteroxanthin [27–30]. We are therefore confident in confirming this xanthophyll to be heteroxanthin.

Xanthophylls diadinoxanthin and violaxanthin, as well as β-carotene, were previously reported in *G*. *semen*, [22, 23]. Violaxanthin and β-carotene, however, were probably only present in concentrations below the detection limit in many of our strains. Alloxanthin was first identified in *G*. *semen* by Sassenhagen et al. [23], previously only found as a major pigment in Cryptophyceae [24]. We confirmed this major accessory pigment in *G*. *semen* by using HPLC-PDA analysis with a reference standard. Alloxanthin was present in every strain as the second major pigment in relation to chlorophyll *a*, following diadinoxanthin.

### Heteroxanthin as a pigment biomarker for *G*. *semen*

Heteroxanthin has previously been identified by complex chemical analysis, and mainly found in very low amounts, or combined with other pigments [22, 27–30, 33]. There is no commercial standard available for heteroxanthin, which is a hydrophilic, polar compound. Thus, a suitable method must successfully separate this pigment peak from compounds with similar properties. This paper presents an adjusted HPLC-PDA method well adapted for separating several polar pigments, including heteroxanthin. This method was successful for cultures of several different algae species in addition to detecting heteroxanthin in lake sediment samples.

In order to use a specific pigment as a biomarker, one must be careful about the specificity of the given pigment [17]. Heteroxanthin is not specific for *G*. *semen*, or even Raphidophyceae [25–30], as confirmed by our results. Among the algae classes containing heteroxanthin, Raphidophyceae (*G*. *semen*) is the most widespread group and the only one with known frequent mass occurrences in Norwegian lakes [6, 35]. *Vaucheria spp*. and *Botrydium* spp. are both terrestrial species, while *Tribonema* spp. are benthic, hence none of these Xanthophyceae are likely to exist in large quantities in lake pelagic or sediments. *Euglena* spp. are often found in freshwater, however only in minor volumes in Lundebyvann and Norway in general [35]. The relevant species of Phaeothamniophyceae have not been detected in Norwegian lakes except for Stichogloea doederleinii [35]. This species is frequently reported in Norwegian phytoplankton samples, however only in modest amounts, and is not observed in Lundebyvann [35]. The unidentified strain NIVA-85/9 was isolated from a freshwater influenced fjord on Svalbard, which reduces the likelihood that this species occurs in or dominates Norwegian lakes. Therefore, we conclude that heteroxanthin is suitable as biomarker for studying *G*. *semen* in Norway. This biomarker is likely also applicable in other boreal lakes. However, an assessment of potentially dominating species containing heteroxanthin should be performed for each area.

Pigments are subject to degradation in the water column upon cell death, at the sediment surface after deposition, and also after burying in the sediments, especially when oxygen is present [16]. The rate of degradation depends on several environmental factors within the lake, however it also depends on the chemical structure and properties of the pigment [16]. The degradability of heteroxanthin is not known, hence it was important to establish whether detection of buried heteroxanthin in lake sediments was possible. By using this modified HPLC-PDA method, we were able to detected heteroxanthin in lake sediments formed 60 years ago. Heteroxanthin was detected as a separate peak in sediments from lake Lundebyvann up to 50 years of age. In older sediments, breakdown products of chlorophyll *a* to some extent interfered with the detection and quantification of heteroxanthin. However, with some attention to absorption spectra, the analysis of heteroxanthin was possible even in these samples.

The amount of pigment deposited (mAU*min cm^2^ year^-1^) in Lundebyvann shows an increased concentration towards 2015, especially the last 30 years. This corresponds to the same increase observed by phytoplankton monitoring in this particular lake as well as other Norwegian lakes [6]. The first recording of *G*. *semen* in Lundebyvann was at the first monitoring survey in 1982 [2]. Recent paleolimnological studies, however, suggest presence from 1977 [36]. An even earlier detection of heteroxanthin by our study suggests the presence of *G*. *semen* in Lake Lundebyvann already in 1957 (+/-13). *G*. *semen* often dominates the phytoplankton community in Lundebyvann for most of the growth season (June-September), with simultaneously high chlorophyll *a* measurements [37] [35, 38]. Thus, most of this chlorophyll *a* is likely to originate from *G*. *semen*. In our study, chlorophyll *a* was quantified as the sum of the native compound and its breakdown products. This largely eliminates the impact of post-deposition breakdown on the chlorophyll *a* measurements, since the main degradation product, Pheophytin-*a*, is known to be extremely stable in sediments [14]. Thus, if the sedimentary heteroxanthin record reflects the historical development of *G*. *semen* in the lake rather than post-depositional breakdown processes in the sediment, we expected similar trends for the concentration of heteroxanthin and of chlorophyll *a*, which is what we found. We therefore conclude that heteroxanthin might be a suitable biomarker for paleolimnological studies using sediment cores. In this study, we isolated and identified xanthophyll pigment heteroxanthin in *G*. *semen* cultures by using a modified HPLC-PDA method based on Wright et al. [32] and LC-MS. This pigment was detectable and stable in lake sediments buried for 60 years, and sufficiently specific to this species.

## Supporting information

S1 Table Strains of algae analyzed by high performance liquid chromatography-photometric diode array to detect heteroxanthin. The table lists all strains analyzed by HPLC-PDA in order to detect heteroxanthin in species common in phytoplankton. The taxonomy is based on the Norwegian Culture Collection of Algae (NORCCA) and AlgaeBase [39].
(DOCX)

S2 Table Pigment to chlorophyll *a* ratios in *Gonyostomum semen* strains. The amount of the most important pigments in relation to chlorophyll a for all G. semen strains analyzed by high performance liquid chromatography-photometric diode array.
(DOCX)

S3 Table Detection of heteroxanthin in sediment core samples from lake Lundebyvann. The table lists all samples were heteroxanthin was detected, including detection parameters retention time and similarity of the absorption spectrum to that of heteroxanthin. Similarity was calculated by the Dionex^TM^ Chromeleon^TM^ software version 7.2.6 (Thermo Scientific^TM^) based on absorption spectrum of heteroxanthin in *G*. *semen* cultures.
(DOCX)

S1 Fig HPLC-PDA chromatogram of sediment sample from lake Lundebyvann. The x-axis shows the retention time (min) and the y-axis the absorbance units (mAU*min). 1 g of dryweight was extracted from the sample which was at depth 13 cm, appr. 51 years of age. All identified peaks are marked with pigment name and retention time. Heteroxanthin is located at 7,9 min.
(TIF)

S2 Fig Absorption spectrum for heteroxanthin in lake sediments. The absorption spectrum is from the 13 cm deep sample, corresponding to appr. age 51 years. Red circles mark the absorption maxima known for heteroxanthin. At this sediment depth, the pigment was influenced by a degradation product of chlorophyll *a*.
(TIF)

## References

[1] Sörensen I. Gonyostomum semen (Ehrenb.) Diesing—en vattenorganism av teoretiskt och praktickt intresse. Svensk Faunistisk Revy. 1954;2:6.

[2] Bjørndalen K, Løvstad Ø. En regionalundersøkelse av innsjøer i Østfold. Eutrofiering og problemalger. VANN. 1984;1:10.

[3] Lepistö L, Antikainen S, Kivinen J. The occurrence of *Gonyostomum semen* (Ehr.) Diesing in Finnish lakes. Hydrobiologia. 1994;273:1–8.

[4] Cronberg G, Lindmark G, Björk S. Mass development of the flagellate *Gonyostomum semen* (Raphidophyta) in Swedish forest lakes—an effect of acidification? Hydrobiologia. 1988;161:217–36.

[5] Hongve D, Løvstad Ø, Bjørndalen K. *Gonyostomum semen*—a new nuisance to bathers in Norwegian lakes. Verh Internat Verein Limnol. 1988;23:430–4.

[6] Hagman CHC, Ballot A, Hjermann DO, Skjelbred B, Brettum P, Ptacnik R. The occurrence and spread of Gonyostomum semen (Ehr.) Diesing (Raphidophyceae) in Norwegian lakes. Hydrobiologia. 2015;744(1):1–14. 10.1007/s10750-014-2050-y WOS:000346182100001.

[7] Rengefors K, Weyhenmeyer GA, Bloch I. Temperature as a driver for the expansion of the microalga *Gonyostomum semen* in Swedish lakes. Harmful Algae. 2012;18:65–73. Epub 25. April 2012.

[8] Lebret K, Fernandez MF, Hagman CHC, Rengefors K, Hansson LA. Grazing resistance allows bloom formation and may explain invasion success of Gonyostomum semen. Limnology and Oceanography. 2012;57(3):727–34. 10.4319/lo.2012.57.3.0727 WOS:000306239300005.

[9] Lebret K, Ostman O, Langenheder S, Drakare S, Guillemette F, Lindstrom ES. High abundances of the nuisance raphidophyte Gonyostomum semen in brown water lakes are associated with high concentrations of iron. Sci Rep. 2018;8:10 10.1038/s41598-017-18422-7 WOS:000444022800067. 30194445 PMC6128840

[10] Trigal C, Hallstan S, Johansson KSL, Johnson RK. Factors affecting occurrence and bloom formation of the nuisance flagellate *Gonyostomum semen* in boreal lakes. harmful Algae. 2013;27:8.

[11] Findlay DL, Paterson MJ, Hendzel LL, Kling HJ. Factors influencing *Gonyostomum semen* blooms in a small boreal reservoir lake. Hydrobiologia. 2005;533:243–52.

[12] Johansson KSL, Luhrig K, Klaminder J, Rengefors K. Development of a quantitative PCR method to explore the historical occurrence of a nuisance microalga under expansion. Harmful Algae. 2016;56:67–76. 10.1016/j.hal.2016.04.012 WOS:000379277100007. 28073497

[13] Hobaek A, Lovik JE, Rohrlack T, Moe SJ, Grung M, Bennion H, et al Eutrophication, recovery and temperature in Lake Mjosa: detecting trends with monitoring data and sediment records. Freshwater Biology. 2012;57(10):1998–2014. 10.1111/j.1365-2427.2012.02832.x WOS:000308405300003.

[14] Reuss NS, Anderson NJ, Fritz SC, Simpson GL. Responses of microbial phototrophs to late-Holocene environmental forcing of lakes in south-west Greenland. Freshwater Biology. 2013;58(4):690–704. 10.1111/fwb.12073 WOS:000316286700006.

[15] Engels S, van Oostrom R, Cherli C, Dungait JAJ, Jansen B, van Aken JM, et al Natural and anthropogenic forcing of Holocene lake ecosystem development at Lake Uddelermeer (The Netherlands). J Paleolimn. 2018;59(3):329–47. 10.1007/s10933-017-0012-x WOS:000425130600003.

[16] Leavitt PR, Hodgson DA. Sedimentary pigments In: Smol JP, Birks HJB, Kast WM, editors. Tracking Environmental Change Using Lake Sediments: Terrestrial, Algal, and Siliceous Indicators. 3. Dordrecht, The Netherlands: Kluwer Academic Publishers; 2001 p. 295–325.

[17] Millie DF, Paerl HW, Hurley JP. Microalgal pigment assessments using High-Performance Liquid-Chromatography—A synopsis of organismal and ecological applications. Can J Fish Aquat Sci. 1993;50(11):2513–27. 10.1139/f93-275 WOS:A1993MZ45600023.

[18] Ellegaard M, Clarke AL, Reuss N, Drew S, Weckstrom K, Juggins S, et al Multi-proxy evidence of long-term changes in ecosystem structure in a Danish marine estuary, linked to increased nutrient loading. Estuar Coast Shelf Sci. 2006;68(3–4):567–78. 10.1016/j.ecss.2006.03.013 WOS:000238871700020.

[19] Gieskes WWC, Kraay GW. Dominance of Cryptophyceae during the phytoplankton spring bloom in the central North-Sea detected by HPLC analysis of pigments. Mar Biol. 1983;75(2–3):179–85. 10.1007/bf00406000 WOS:A1983RG96900009.

[20] Chapman DJ, Haxo F. Chloroplast pigments of Chloromonadophyceae. Journal of Phycology. 1966;2(2):89–91. 10.1111/j.1529-8817.1966.tb04599.x 27053320

[21] Guillard RRL, Lorenzen CJ. Yellow-green algae with chlorophyllide c. J Phycol. 1972;8:10–4.

[22] Fiksdahl A, Withers N, Guillard RRL, Liaaenjensen S. Carotenoids of the Raphidophyceae—a chemosystematic contribution. Comp Biochem Physiol B-Biochem Mol Biol. 1984;78(1):265–71. 10.1016/0305-0491(84)90181-0 WOS:A1984SV19800046.

[23] Sassenhagen I, Rengefors K, Richardson TL, Pinckney JL. Pigment composition and photoacclimation as keys to the ecological success of *Gonyostomum semen* (Raphidophyceae, Stramenopiles). J Phycol. 2014;50:9. 10.1111/jpy.12246 26988794

[24] Chapman DJ. Three new carotenoids isolated from algae. Phytochemistry. 1966;5(6):1331–3. 10.1016/S0031-9422(00)86131-2.

[25] Kleinig H, Egger K. Carotinoide der Vaucheriales Vaucheria und Botrydium (Xanthophyceae). Zeitschrift Fur Naturforschung Part B-Chemie Biochemie Biophysik Biologie Und Verwandten Gebiete. 1967;B 22(8):868–&. WOS:A19679866500015. 4384765

[26] Strain HH, Svec WA, Aitzetmuller K, Grandolfo M, Katz JJ. Molecular weights and empirical formulas of Xanthophylls of Vaucheria. Phytochemistry. 1968;7(8):1417–+. 10.1016/s0031-9422(00)85649-6 WOS:A1968B447400034.

[27] Strain HH, Benton FL, Grandolfo MC, Aitzetmueller K, Svec WA, Katz JJ. Heteroxanthin, diatoxanthin and diadinoxanthin from *Tribonema aequale*. Phytochemistry. 1970;9(12):2561–+. 10.1016/s0031-9422(00)85778-7 WOS:A1970I161800022.

[28] Nitsche H. Heteroxanthin in Euglena gracilis. Arch Mikrobiol. 1973;90(2):151–5. 10.1007/bf00414517 4196487

[29] Andersen RA, Potter D, Bidigare RR, Latasa M, Rowan K, O'Kelly CJ. Characterization and phylogenetic position of the enigmatic golden alga Phaeothamnion confervicola: Ultrastructure, pigment composition and partial SSU rDNA sequence. Journal of Phycology. 1998;34(2):286–98. 10.1046/j.1529-8817.1998.340286.x WOS:000073239700012.

[30] Bailey JC, Bidigare RR, Christensen SJ, Andersen RA. Phaeothamniophyceae classis nova: A new lineage of chromophytes based upon photosynthetic pigments, rbcL sequence analysis and ultrastructure. Protist. 1998;149(3):245–63. 10.1016/S1434-4610(98)70032-X WOS:000076195000005. 23194637

[31] Xiao Y, Rohrlack T, Riise G. Unraveling long-term changes in lake color based on optical properties of lake sediment. Science of The Total Environment. 2019:134388 10.1016/j.scitotenv.2019.134388. 33736194

[32] Wright SW, Jeffrey SW, Mantoura RFC, Llewellyn CA, Bjornland T, Repeta D, et al Improved HPLC method for the analysis of chlorophylls and carotenoids from marine phytoplankton. Mar Ecol-Prog Ser. 1991;77(2–3):183–96. 10.3354/meps077183 WOS:A1991GV75600008.

[33] Buchecker R, Liaaen-Jensen S. Absolute configuration of heteroxanthin and diadinoxanthin. Phytochemistry. 1977;16(6):729–33. 10.1016/s0031-9422(00)89242-0 WOS:A1977DF76600024.

[34] Guaratini T, Vessecchi RL, Lavarda FC, Campos P, Naal Z, Gates PJ, et al New chemical evidence for the ability to generate radical molecular ions of polyenes from ESI and HR-MALDI mass spectrometry. Analyst. 2004;129(12):1223–6. 10.1039/b412154f WOS:000225335500012. 15565222

[35] Norwegian Environment Agency. Vannmiljø [26.07.2019]. Available from: https://vannmiljo.miljodirektoratet.no.

[36] Rohrlack T, Haaland S. Paleolimnologisk undersøkelse av Lundebyvannet i Eidsberg kommune. Ås, Norway: Norwegian University of Life Sciences, 2017.

[37] Haande S, Edvardsen H, Eriksen T, Kile M, Hagman CHC, Borch H, et al Tilstandsklassifisering av vannforekomster i Vannområde Glomma Sør for Øyeren (2011) i henhold til vannforskriften. Norwegian Institute for Water Research: 2012.

[38] Stabell T. Klassifisering av innsjøer i Vannområde Glomma sør for Øyeren etter kvalitetselementet «planteplankton» Datarapport, 2018. FAUN, 2019.

[39] AlgaeBase. algaebase.org [02.06.2019]. Available from: http://www.algaebase.org/.

