# Supplementary material for: Heteroxanthin as a pigment biomarker for Gonyostomum semen (Raphidophyceae)
Source: PLoS One. 2019 Dec 18;14(12):e0226650. doi: 10.1371/journal.pone.0226650 (PMC6919615; doi:10.1371/journal.pone.0226650)
Supplement: S1 Table — The table lists all strains analyzed by HPLC-PDA in order to detect heteroxanthin in species common in phytoplankton. The taxonomy is based on the Norwegian Culture Collection of Algae (NORCCA) and AlgaeBase [39]. (DOCX) [file pone.0226650.s001.docx]

**S1 Table. Strains of algae analyzed by high performance liquid chromatography-photometric diode array to detect heteroxanthin.**

| **Class** | **Strain number** | **Genus, species** | **Origin** |
| --- | --- | --- | --- |
| Bacillariophyceae | NIVA-BAC 3^a^ | *Asterionella formosa* | United Kingdom |
|  | NIVA-BAC 18^a^ | *Fragilaria rumpens* | Lake Naivasha, Kenya |
| Chlorophyceae | NIVA-CHL 21 | *Chlamydomonas reinhardtii* | Spydeberg, Østfold, Norway |
|  | NIVA-CHL 25 | *Chlamydomonas noctigama* | Norway |
|  | NIVA-CHL 7 | *Desmodesmus communis* | Lake Årungen, Norway |
|  | NIVA-CHL 60^a^ | *Monoraphidium dybowski* | Lake Östra Nedsjön, Sverige |
|  | NIVA-CHL 74 | *Monoraphidium cf.minutum* | Kenya |
|  | NIVA-CHL 8 | *Monoraphidium griffithii* | Lake Årungen, Norway |
|  | K-1612^a^ | *Desmodesmus armatus* | Lac de Thorenc, France |
|  | NIVA-CHL 133 | *Scenedesmus sp.* | Norway |
|  | NIVA-CHL 77 | *Sphaerocystis schroeterii* | Lake Turkana, Kenya |
| Chrysophyceae | NIVA-5/14^a^ | *Dinobryon cf. sertularia* | Lake Sannes-Langen, Norge |
|  | NIVA-85/9 | *Unidentified chrysophycean* | Ripjfjorden, Spitsbergen, Norway |
| Conjugatophyceae | NIVA-CHL 150 | *Closterium sp.* | Bærum, Norway |
|  | NIVA-CHL 185 | *Cosmarium sp.* | Lake Digerruddammen, Norway |
|  | NIVA-CHL 4 | *Staurastrum gracile* | Lake Gjersjøen, Norway |
|  | NIVA-CHL 49 | *Staurastrum sp.* | Lake Haugatjønn, Norway |
| Cryptophyceae | NIVA-2/81 | *Cryptomonas pyrenoidifera* | Lake Gjersjøen, Norway |
|  | NIVA-3/81 | *Cryptomonas rostratiformis* | Lake Helgetjernet, Norway |
|  | NIVA-3/09 | *Cryptomonas sp.* | Kvernhusbekken, Norway |
|  | NIVA-1/10 | *Cryptomonas sp.* | Norway |
|  | NIVA-8/82 | *Rhodomonas lacustris* | Nordbytjernet, Norway |
| Cyanophyceae | NIVA-CYA 269/2 | *Dolichospermum flos-aquae* | Lake Frøylandsvatnet, Norway |
|  | NIVA-CYA 850 | *Anabaena planctonica* | Lake Kolbotnvatnet, Norway |
|  | NIVA-CYA 226/2 | *Dolichospermum spiroides* | Lake Holstadvatnet, Norway |
|  | NIVA-CYA 851 | *Aphanizomenon gracile* | Sundbyfosvannet, Norway |
|  | NIVA-CYA 474 | *Aphanocapsa muscicola* | Vikedal, Norway |
|  | NIVA-CYA 16 | *Merismopedia punctata* | Lake Steinsfjorden, Norway |
|  | NIVA-CYA 161/1 | *Microcystis botrys* | Lake Mosvatnet, Norway |
|  | NIVA-CYA 228/4 | *Microcystis aeruginosa* | Lake Akersvatnet, Norway |
|  | NIVA-CYA 607^a^ | *Woronichinia naegeliana* | Akersvannet, Norway |
|  | NIVA-CYA 612^a^ | *Woronichinia naegeliana* | Steinsfjorden, Norway |
| Dinophyceae | NIVA-1/13^a^ | *Peridinium cf. cinctum* | Kindrogan Pond, Scotland |
| Euglenophyceae | NIVA-1/79^b^ | *Euglena gracilis* | Unknown |
|  | NIVA-11/91 | *Euglena sp.* | Lake Kalvsjøtjernet, Norway |
|  | K-1464 | *Euglena sanguinea* | Denmark |
|  | K-1465 | *Euglena sanguinea* | Denmark |
|  | K-1380 | *Trachelomonas sp.* | Seealpsee, Switzerland |
| Mediophyceae | NIVA-BAC 74^a^ | *Cyclotella sp.* | Sundbyfosvannet, Norway |
| Phaeothamniophyceae | K-1003 | *Phaeothamnion sp.* | Pond, Copenhagen, Denmark |
|  | K-1186^c^ | *Phaeothamnion confervicola* | Unknown |
| Raphidophyceae | NIVA-2/13 | *Vacuolaria virescens* | Lake Skjærsjøen |
|  | NIVA-2/14^a^ | *Vacuolaria virescens* | Sannes-Langen, Norway |
|  | NIVA-3/14 | *Vacuolaria virescens* | Sannes-Langen, Norway |
|  | NIVA-4/14 | *Vacuolaria virescens* | Sannes-Langen, Norway |
|  | NIVA-1/15^d^ | *Vacuolaria virescens* | Cheshire, UK |
| Synurophyceae | NIVA-5/09 | *Synura sp.* | Lake Adalstjern, Norway |
|  | K-1875^a^ | *Synura petersenii* | Lake Kyynäröjärvi, Finland |
| Trebouxiophyceae | NIVA-CHL 87 | *Botryococcus cf. braunii* | Lake Munkedamsvatnet, Norway |
|  | NIVA-CHL 15 | *Chlorella sp.* | Spydeberg, Norway |
|  | NIVA-CHL 19 | *Chlorella vulgaris* | Spydeberg, Norway |
|  | NIVA-CHL 187 | *Dictyosphaerium sp.* | Lake Askjemvannet, Norway |
|  | NIVA-CHL 42 | *Dictyosphaerium pulchellum var. minutum* | Lake Naivasha, Kenya |
|  | NIVA-CHL 119 | *Koliella longispina* | Lake Stordammen, Norway |
| Xanthophyceae | K-0162 | *Tribonema minus* | Fen at Sandbjerg, Denmark |
|  | K-0173 | *Tribonema regulare* | Pøleåen, Arresø, Denmark |
|  | K-0087 | *Tribonema aequale* | Denmark |

The table lists all strains analyzed by HPLC-PDA in order to detect heteroxanthin in species common in phytoplankton. The taxonomy is based on the Norwegian Culture Collection of Algae (NORCCA) and AlgaeBase.

^a^Strains with no detectable pigments

^b^Synonym strains: CCAP 1224/5Z, SAG 1224-5/25, UTEX 753, ATCC 12894, UTCC 95

^c^Synonym strains: CCMP 637, SAG 119.79, A-7741

^d^Synonym strain: SAG 1195-1
