# Supplementary material for: Heteroxanthin as a pigment biomarker for Gonyostomum semen (Raphidophyceae)
Source: PLoS One. 2019 Dec 18;14(12):e0226650. doi: 10.1371/journal.pone.0226650 (PMC6919615; doi:10.1371/journal.pone.0226650)
Supplement: S2 Table — The amount of the most important pigments in relation to chlorophyll a for all G. semen strains analyzed by high performance liquid chromatography-photometric diode array. (DOCX) [file pone.0226650.s002.docx]

**S2 Table. Pigment to chlorophyll *a* ratios in *Gonyostomum semen* strains.**

| **Strain No.** | **Hetero-xanthin** | **Viola-xanthin** | **Diadino-xanthin** | **Diato-xanthin** | **Alloxanthin** | **β-carotene** | **Chlorophyll *c2* derivate** |
| --- | --- | --- | --- | --- | --- | --- | --- |
| **NIVA-7/05** | 0.046 | 0.008 | 0.308 | 0.000 | 0.137 | 0.034 | 0.005 |
| **NIVA-2/09** | 0.062 | 0.000 | 0.285 | 0.000 | 0.123 | 0.000 | 0.000 |
| **NIVA-2/10** | 0.048 | 0.000 | 0.280 | 0.000 | 0.234 | 0.010 | 0.000 |
| **NIVA-5/13** | 0.035 | 0.004 | 0.335 | 0.000 | 0.119 | 0.027 | 0.004 |
| **NIVA-6/13** | 0.073 | 0.000 | 0.319 | 0.000 | 0.338 | 0.000 | 0.000 |
| **NIVA-10/13** | 0.058 | 0.000 | 0.367 | 0.000 | 0.181 | 0.000 | 0.000 |
| **NIVA-11/13** | 0.048 | 0.000 | 0.308 | 0.000 | 0.163 | 0.000 | 0.000 |
| **NIVA-12/13** | 0.059 | 0.000 | 0.304 | 0.000 | 0.143 | 0.000 | 0.000 |
| **NIVA-13/13** | 0.043 | 0.007 | 0.263 | 0.000 | 0.157 | 0.012 | 0.000 |
| **NIVA-15/13** | 0.072 | 0.000 | 0.249 | 0.000 | 0.167 | 0.000 | 0.000 |
| **NIVA-16/13** | 0.044 | 0.000 | 0.334 | 0.000 | 0.244 | 0.010 | 0.000 |
| **NIVA-17/13** | 0.047 | 0.003 | 0.221 | 0.000 | 0.218 | 0.004 | 0.000 |
| **NIVA-18/13** | 0.046 | 0.000 | 0.300 | 0.000 | 0.115 | 0.000 | 0.000 |
| **NIVA-24/13** | 0.048 | 0.000 | 0.248 | 0.000 | 0.165 | 0.000 | 0.000 |
| **NIVA-33/13** | 0.026 | 0.003 | 0.289 | 0.000 | 0.110 | 0.000 | 0.004 |
| **NIVA-34/13** | 0.039 | 0.000 | 0.223 | 0.000 | 0.153 | 0.000 | 0.000 |
| **K-1835** | 0.045 | 0.000 | 0.196 | 0.000 | 0.099 | 0.000 | 0.000 |
| **NOR 17** | 0.045 | 0.000 | 0.219 | 0.000 | 0.089 | 0.000 | 0.000 |
| **NOR 18** | 0.043 | 0.000 | 0.236 | 0.000 | 0.111 | 0.000 | 0.000 |
| **NOR 19** | 0.036 | 0.000 | 0.203 | 0.046 | 0.059 | 0.000 | 0.000 |
| **NOR 20** | 0.031 | 0.005 | 0.202 | 0.013 | 0.095 | 0.010 | 0.000 |
| **NOR 21** | 0.043 | 0.000 | 0.207 | 0.000 | 0.087 | 0.000 | 0.000 |
| **NOR 22** | 0.032 | 0.000 | 0.208 | 0.000 | 0.077 | 0.000 | 0.000 |
| **NOR 23** | 0.048 | 0.000 | 0.220 | 0.000 | 0.094 | 0.000 | 0.000 |
| **NOR 24** | 0.039 | 0.003 | 0.197 | 0.000 | 0.110 | 0.007 | 0.000 |
| **NOR 25** | 0.034 | 0.000 | 0.198 | 0.000 | 0.069 | 0.005 | 0.000 |
| **NOR 26** | 0.032 | 0.000 | 0.185 | 0.000 | 0.075 | 0.000 | 0.000 |
| **NOR 27** | 0.031 | 0.000 | 0.187 | 0.038 | 0.051 | 0.000 | 0.000 |

The amount of the most important pigments in relation to chl-a for all *G. semen* strains analyzed by high performance liquid chromatography-photometric diode array.
