# Supplementary material for: Heteroxanthin as a pigment biomarker for Gonyostomum semen (Raphidophyceae)
Source: PLoS One. 2019 Dec 18;14(12):e0226650. doi: 10.1371/journal.pone.0226650 (PMC6919615; doi:10.1371/journal.pone.0226650)
Supplement: S3 Table — The table lists all samples were heteroxanthin was detected, including detection parameters retention time and similarity of the absorption spectrum to that of heteroxanthin. Similarity was calculated by the DionexTM ChromeleonTM software version 7.2.6 (Thermo ScientificTM) based on absorption spectrum of heteroxanthin in G. semen cultures. (DOCX) [file pone.0226650.s003.docx]

**S3 Table. Detection of heteroxanthin in sediment core samples from lake Lundebyvann**

| **Sample depth (cm)** | **Amount extracted**  **(mg dryweight)** | **Peak retention time**  **(min)** | **Peak designation** | **Similarity**  **(%)** |
| --- | --- | --- | --- | --- |
| 1 | 250 | 7,9 | Heteroxanthin | 99,97 |
| 2 | 250 | 7,9 | Heteroxanthin | 99,42 |
| 3 | 250 | 7,9 | Heteroxanthin | 99,91 |
| 4 | 250 | 7,9 | Heteroxanthin | 99,82 |
| 5 | 250 | 7,9 | Heteroxanthin | 99,37 |
| 6 | 250 | 7,9 | Heteroxanthin | 98,77 |
| 7 | 250 | 7,9 | Heteroxanthin | 99,88 |
| 8 | 250 | 7,9 | Heteroxanthin | 99,65 |
| 9 | 250 | 7,9 | Heteroxanthin | 99,49 |
| 10 | 250 | 7,9 | Heteroxanthin | 99,55 |
| 11 | 250 | 7,9 | Heteroxanthin | 99,11 |
| 12 | 250 | 7,9 | Heteroxanthin | 96,73 |
| 13 | 1000 | 7,9 | Heteroxanthin | 83,26 |
| 14 | 1000 | 7,9 | Heteroxanthin | 94,65 |
| 15 | 1000 | 7,9 | Heteroxanthin | 91,47 |

The table lists all samples where heteroxanthin was detected, including detection parameters retention time and similarity of the absorption spectrum to that of heteroxanthin. Similarity was calculated by the Dionex^TM^ Chromeleon^TM^ software version 7.2.6 (Thermo Scientific^TM^) based on absorption spectrum of heteroxanthin in *G. semen* cultures.
