# Supplementary figures and images for: Heteroxanthin as a pigment biomarker for Gonyostomum semen (Raphidophyceae)
Source: PLoS One. 2019 Dec 18;14(12):e0226650. doi: 10.1371/journal.pone.0226650 (PMC6919615; doi:10.1371/journal.pone.0226650)

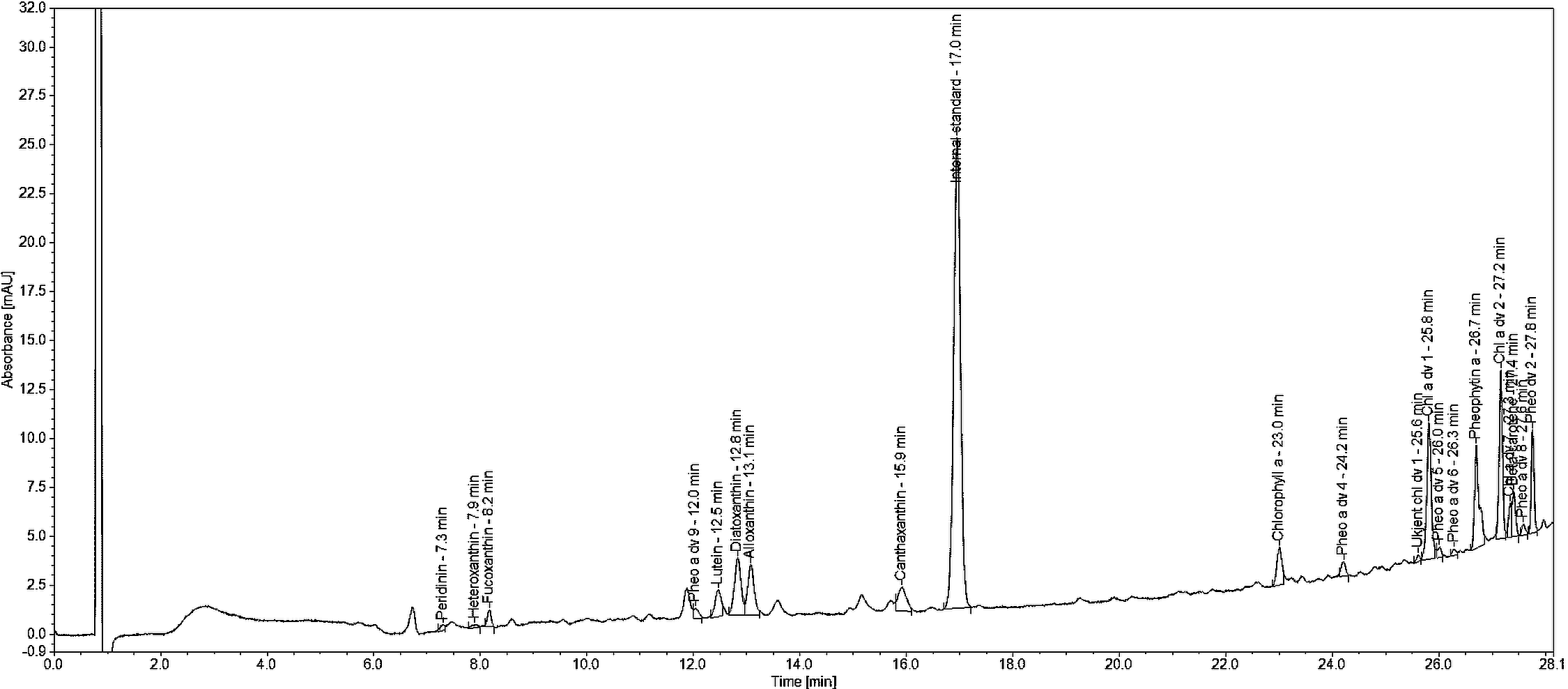

Supplement: S1 Fig — The x-axis shows the retention time (min) and the y-axis the absorbance units (mAU*min). 1 g of dryweight was extracted from the sample which was at depth 13 cm, appr. 51 years of age. All identified peaks are marked with pigment name and retention time. Heteroxanthin is located at 7,9 min. (TIF) [file pone.0226650.s004.tif]

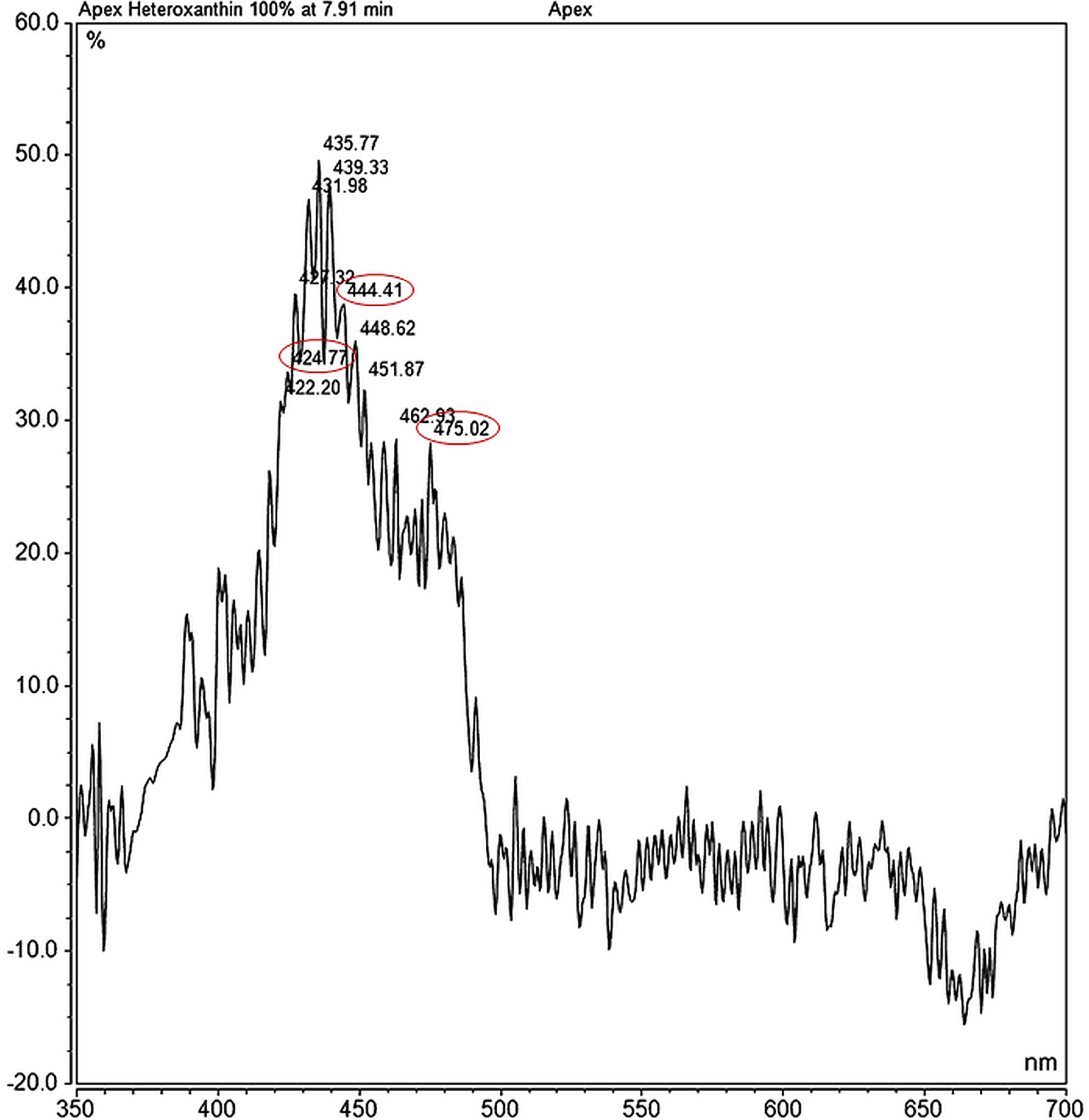

Supplement: S2 Fig — The absorption spectrum is from the 13 cm deep sample, corresponding to appr. age 51 years. Red circles mark the absorption maxima known for heteroxanthin. At this sediment depth, the pigment was influenced by a degradation product of chlorophyll a. (TIF) [file pone.0226650.s005.tif]
